# Supplementary material for: Optimizing hepatitis B diagnosis for mothers in a low-resource setting: A field pilot of Xpert point-of-care viral load testing in Ugandan antenatal clinics
Source: PLOS Glob Public Health. 2026 May 4;6(5):e0006380. doi: 10.1371/journal.pgph.0006380 (PMC13138665; doi:10.1371/journal.pgph.0006380)
Supplement: S1 File — (DOCX) [file pgph.0006380.s001.docx]

## **Supplementary analysis for Phase I analysis**

## **Phase I: Laboratory performance**

The results of the clinical performance evaluation among HBV-positive specimens are summarized in the table below.

Table A. Summary of the results of the clinical performance evaluation

|  |  | **Reference assay (COBAS AmpliPrep-COBAS TaqMan Hepatitis B Virus Quantitative test)** | | | |
| --- | --- | --- | --- | --- | --- |
| **Xpert HBV Viral load** |  | TND | Detected,  < 20 IU/mL | Detected quantified | Detected  >10^9^ IU/Ml |
|  | TND | 11 | 0 | 0 | 0 |
|  | Detected  < LLoQ | 1 | 0 | 0 | 0 |
|  | Detected  quantified | 2 | 0 | 34 | 0 |
|  | Detected  > ULoQ | 0 | 0 | 0 | 0 |

*TND: target not detected; LLoQ : lower limit of quantification; ULoQ: upper limit of quantification (insert numbers)*

## Bland-Altman analysis

Figure A: The Bland-Altman analysis is represented above and the results of the analysis are summarized in table 6.

The results of the analysis are summarized in table B.

**Table B. Results of the Bland-Altman analysis of Xpert® HBV Viral Load on Gene Xpert systems compared to the reference method**

| **Bias**  **(log_10_ cp/mL)** | **Standard deviation (log_10_ cp/mL)** | **Limits of agreement (log_10_ cp/mL)** | |
| --- | --- | --- | --- |
|  |  | **Lower limit (95% CI)** | **Upper limit (95% CI)** |
| 0.06 | 0.25 | -0.43 | 0.56 |

*Note: The Target was not detected, and < LLOQ cp/mL results were excluded from this analysis because they were unquantifiable.*

***Table C: Comparison of all results between Cobas Taqman and Xpert HBV viral load platforms***

|  |  |  | **Cobas Taqman/CAPCTM HBV Viral load** | | **Xpert HBV Viral Load** | |
| --- | --- | --- | --- | --- | --- | --- |
| **S/N** | **Sample ID** | **Date of collection** | **Date of testing** | **Result (IU/Ml)** | **Date of testing** | **Result (IU/Ml)** |
| 1 | V2403-0004/080 | 01/03/2024 | 06/03/2024 | 4,420,000 | 12/03/2024 | 5480000 |
| 2 | V2403-0005/011 | 01/03/2024 | 06/03/2024 | 92600 | 12/03/2024 | 53300 |
| 3 | V2403-0005/064 | 04/03/2024 | 06/03/2024 | 1296000 | 12/03/2024 | 3860000 |
| 4 | V2403-0005/073 | 04/03/2024 | 06/03/2024 | 2440000 | 12/03/2024 | 6930000 |
| 5 | V2403-0005/078 | 04/03/2024 | 06/03/2024 | 17680000 | 12/03/2024 | 17200000 |
| 6 | V2403-0005/089 | 04/03/2024 | 06/03/2024 | 81400 | 12/03/2024 | 36500 |
| 7 | V2403-0005/096 | 04/03/2024 | 07/03/2024 | 0 | 12/03/2024 | 0 |
| 8 | V2403-0005/097 | 04/03/2024 | 07/03/2024 | 66200 | 12/03/2024 | 43700 |
| 9 | V2403-0005/094 | 04/03/2024 | 07/03/2024 | 0 | 12/03/2024 | 26 |
| 10 | V2403-0006/006 | 06/03/2024 | 07/03/2024 | 0 | 12/03/2024 | 0 |
| 11 | V2403-0006/007 | 06/03/2024 | 07/03/2024 | 0 | 12/03/2024 | 0 |
| 12 | V2403-0006/010 | 05/03/2024 | 07/03/2024 | 892000 | 12/03/2024 | 1140000 |
| 13 | V2403-0006/016 | 03/03/2024 | 07/03/2024 | 0 | 12/03/2024 | Invalid |
| 14 | V2403-0006/020 | 05/03/2024 | 07/03/2024 | 0 | 12/03/2024 | 0 |
| 15 | V2403-0006/057 | 06/03/2024 | 07/03/2024 | 43600000 | 12/03/2024 | 93900000 |
| 16 | V2403-0006/074 | 05/03/2024 | 07/03/2024 | 904000 | 12/03/2024 | 1100000 |
| 17 | V2403-0006/034 | 06/03/2024 | 07/03/2024 | 0 | 13/03/2024 | 0 |
| 18 | V2403-0006/035 | 01/03/2024 | 07/03/2024 | 0 | 13/03/2024 | 118 |
| 19 | V2403-0006/039 | 04/03/2024 | 07/03/2024 | 0 | 13/03/2024 | 0 |
| 20 | V2403-0006/067 | 06/03/2024 | 07/03/2024 | 0 | 13/03/2024 | 0 |
| 21 | V2403-0006/070 | 05/03/2024 | 07/03/2024 | 0 | 13/03/2024 | 0 |
| 23 | V2403-0006/086 | 01/03/2024 | 11/03/2024 | 48000000 | 13/03/2024 | 64400000 |
| 24 | V2403-0007/022 | 05/03/2024 | 11/03/2024 | 14600000 | 13/03/2024 | 22000000 |
| 25 | V2403-0007/063 | 07/03/2024 | 11/03/2024 | 222000000 | 13/03/2024 | 325000000 |
| 26 | V2403-0008/004 | 04/03/2024 | 11/03/2024 | 0 | 13/03/2024 | 1 |
| 27 | V2403-0008/006 | 07/03/2024 | 11/03/2024 | 9200000 | 13/03/2024 | 14000000 |
| 28 | V2403-0008/011 | 06/03/2024 | 11/03/2024 | 0 | 13/03/2024 | 0 |
| 29 | V2403-0008/014 | 08/02/2024 | 11/03/2024 | 0 | 13/03/2024 | 0 |
| 30 | V2403-0008/029 | 07/03/2024 | 11/03/2024 | 320000000 | 13/03/2024 | 253000000 |
| 31 | V2403-0008/042 | 26/02/2024 | 11/03/2024 | 1686000 | 13/03/2024 | 1760000 |
| 32 | V2403-0006/090 | 04/03/2024 | 13/03/2024 | 28000 | 14/03/2024 | 16800 |
| 33 | V2403-0007/001 | 08/03/2024 | 13/03/2024 | 37000 | 14/03/2024 | 41600 |
| 34 | V2403-0007/043 | 04/03/2024 | 13/03/2024 | 198600 | 14/03/2024 | 163000 |
| 35 | V2403-0007/047 | 06/03/2024 | 13/03/2024 | 29400 | 14/03/2024 | 19100 |
| 36 | V2403-0007/060 | 07/03/2024 | 13/03/2024 | 24400 | 14/03/2024 | 36800 |
| 37 | V2403-0008/009 | 05/03/2024 | 13/03/2024 | 45800 | 14/03/2024 | 46000 |
| 38 | V2403-0008/018 | 07/03/2024 | 13/03/2024 | 197800 | 14/03/2024 | 220000 |
| 39 | V2403-0008/028 | 28/02/2024 | 13/03/2024 | 0 | 14/03/2024 | 0 |
| 40 | V2403-0008/058 | 07/03/2024 | 13/03/2024 | 5940000 | 14/03/2024 | 12700000 |
| 41 | V2403-0008/068 | 07/03/2024 | 13/03/2024 | 154000000 | 14/03/2024 | 173000000 |
| 42 | V2403-0009/004 | 01/03/2024 | 13/03/2024 | 36600 | 14/03/2024 | 28900 |
| 43 | V2403-0009/005 | 23/02/2024 | 13/03/2024 | 74000 | 14/03/2024 | 173000 |
| 44 | V2403-0009/012 | 09/03/2024 | 13/03/2024 | 608000 | 14/03/2024 | 1300000 |
| 45 | V2403-0009/013 | 09/03/2024 | 13/03/2024 | 16000000 | 14/03/2024 | 109000000 |
| 46 | V2403-0009/089 | 12/03/2024 | 13/03/2024 | 28400 | 14/03/2024 | 18200 |
| 47 | V2403-0009/093 | 04/03/2024 | 13/03/2024 | 195400 | 14/03/2024 | 98600 |
| 48 | V2403-010/041 | 12/03/2024 | 13/03/2024 | 117200 | 14/03/2024 | 96500 |
| 49 | V2403-010/087 | 10/03/2024 | 13/03/2024 | 50000 | 14/03/2024 | 33300 |
| 50 | V2403-010/098 | 11/03/2024 | 13/03/2024 | 68000 | 14/03/2024 | 63700 |

## Correlation

The Deming regression analysis of Xpert® HBV Viral Load on Gene Xpert systems and the COBAS AmpliPrep-COBAS TaqMan Hepatitis B Virus Quantitative test is represented in figure 2. Un quantifiable results were excluded from the analysis.

Figure B: Deming regression analysis of Xpert® HBV Viral Load on Gene Xpert systems and the COBAS AmpliPrep-COBAS TaqMan Hepatitis B Virus Quantitative test.
