# Supplementary material for: Optimizing hepatitis B diagnosis for mothers in a low-resource setting: A field pilot of Xpert point-of-care viral load testing in Ugandan antenatal clinics
Source: PLOS Glob Public Health. 2026 May 4;6(5):e0006380. doi: 10.1371/journal.pgph.0006380 (PMC13138665; doi:10.1371/journal.pgph.0006380)
Supplement: S2 File — (DOCX) [file pgph.0006380.s002.docx]

## ***Maternal Sociodemographic characteristics and other relevant background information***

*Table 1: Maternal Socio-Demographic Information*

| **Variables** | | **Frequency (N=188)** | **%** |
| --- | --- | --- | --- |
| **Maternal Information** | | | |
| Age Category | 15-19 | 1 | 0.5 |
|  | 20-24 | 60 | 31.9 |
|  | 25-29 | 62 | 33.0 |
|  | 30-34 | 45 | 23.9 |
|  | 35-39 | 16 | 8.5 |
|  | 40-44 | 2 | 1.1 |
|  | Missing data | 2 | 1.1 |
| Gravidity | Gravida 1 | 39 | 20.7 |
|  | Gravida 2 | 42 | 22.3 |
|  | Gravida 3 | 35 | 18.6 |
|  | Gravida 4 | 27 | 14.4 |
|  | Gravida 5+ | 32 | 17.0 |
|  | Missing data | 13 | 6.9 |
| Antenatal attendance | ANC 1 | 38 | 20.2 |
|  | ANC 2 | 39 | 20.7 |
|  | ANC 3 | 39 | 20.7 |
|  | ANC 4 | 26 | 13.8 |
|  | ANC 5+ | 33 | 17.6 |
|  | Missing data | 13 | 6.9 |
| Number of children | 0 | 30 | 16.0 |
|  | 1 | 32 | 17.0 |
|  | 2 | 39 | 20.7 |
|  | 3 | 40 | 21.3 |
|  | 4 | 13 | 6.9 |
|  | ≥5 | 34 | 18.1 |
| Tested for HIV | No | 3 | 1.6 |
|  | Yes | 185 | 98.4 |
| Tested for syphilis | No | 6 | 3.2 |
|  | Yes | 182 | 96.8 |
| HIV status | Positive | 3 | 1.6 |
|  | Negative | 182 | 96.8 |
|  | Missing data | 3 | 1.6 |
| Mother had known HIV HIV-positive status before this pregnancy | No | 2 | 66.7 |
|  | Yes | 1 | 33.3 |
| Mother on Tenofovir ART regimen for HIV/AIDS | Missing data | 2 | 1.1 |
|  | Yes | 1 | .5 |
| Syphilis status | Positive | 8 | 4.3 |
|  | Negative | 174 | 92.5 |
|  | Missing data | 6 | 3.2 |

*Table 3: Maternal exposure history and household risk factors for HBV*

| **Variables** | | **Frequency (N=188)** | **%** |
| --- | --- | --- | --- |
| **Transmissions and risk factors** | | | |
| History of blood transfusion | No | 170 | 90.4 |
|  | Yes | 12 | 6.4 |
|  | Missing data | 1236 | 3.2 |
| History of general surgical procedures | No | 152 | 80.9 |
|  | Yes | 21 | 11.2 |
|  | Missing data | 15 | 8.0 |
| History of unsafe dental procedures | No | 105 | 55.9 |
|  | Yes | 65 | 34.6 |
|  | Missing data | 18 | 9.6 |
| History of body tattooing | No | 181 | 96.3 |
|  | Yes | 6 | 3.2 |
|  | Missing data | 1 | .5 |
| HBV-positive household member | No | 156 | 83.0 |
|  | Yes | 32 | 17.0 |
| Spouse HBV positive | Yes | 9 | 4.8 |
|  | No | 179 | 95.2 |
| History of a liver disease | No | 175 | 93.1 |
|  | Yes | 13 | 6.9 |
| History of kidney disease | No | 184 | 97.9 |
|  | Yes | 1 | .5 |
|  | Missing data | 3 | 1.6 |
| Smokes | No | 186 | 98.9 |
|  | Yes | 1 | .5 |
|  | Missing data | 1 | .5 |
| Child HBV positive | No | 138 | 73.4 |
|  | Missing data | 20 | 10.6 |
|  | Not applicable?? | 30 | 16.0 |
| Child <18 years living with HIV | No | 129 | 68.6 |
|  | Yes | 9 | 4.8 |
|  | Missing data | 20 | 10.6 |
|  | Not applicable | 30 | 16.0 |
| HIV+ children <18 years started on ART (N=9) | No | 8 | 4.3 |
|  | Yes | 1 | 0.5 |

*Table 4: Partner demographics and testing status for HBV, HIV, and syphilis*

| **Variables** | | **Frequency (N=186)** | **%** |
| --- | --- | --- | --- |
| Age category | 20-24 years | 7 | 3.7 |
|  | 25-29 years | 48 | 25.5 |
|  | 30-34 years | 53 | 28.2 |
|  | 35-39 years | 36 | 19.1 |
|  | 40-44 years | 20 | 10.6 |
|  | 45-49 years | 10 | 5.3 |
|  | 55-59 years | 1 | .5 |
|  | Missing data | 13 | 6.9 |
| Partner tested for HBV | No | 76 | 40.4 |
|  | Yes | 95 | 50.5 |
|  | Missing data | 17 | 9.0 |
| Partner tested for HIV | No | 51 | 27.1 |
|  | Yes | 122 | 64.9 |
|  | Missing data | 15 | 8.0 |
| Partner tested for syphilis | No | 70 | 37.2 |
|  | Yes | 100 | 53.2 |
|  | Missing data | 18 | 9.6 |
| Partner HBV status | Negative | 84 | 44.7 |
|  | Positive | 8 | 4.3 |
|  | Missing data | 20 | 10.6 |
|  | No tested | 76 | 40.4 |
| Partner HIV status | HIV Negative | 112 | 59.5 |
|  | HIV Positive | 2 | 1.1 |
|  | Missing data | 23 | 12.3 |
|  | Not tested | 51 | 27.1 |
| Partner syphilis status | Negative | 95 | 50.5 |
|  | Positive | 3 | 1.5 |
|  | Missing data | 20 | 10.7 |
|  | Not tested | 70 | 37.2 |
