# Supplementary material for: Optimizing hepatitis B diagnosis for mothers in a low-resource setting: A field pilot of Xpert point-of-care viral load testing in Ugandan antenatal clinics
Source: PLOS Glob Public Health. 2026 May 4;6(5):e0006380. doi: 10.1371/journal.pgph.0006380 (PMC13138665; doi:10.1371/journal.pgph.0006380)
Supplement: S3 File — (DOCX) [file pgph.0006380.s003.docx]

***Supplementary material 3: Table showing different time points from sample collection to results receipt by pregnant women (mothers)***

| ***Response***  ***(in days)*** | ***TAT 1 (Sample collection Vs sample dispatch to the Laboratory)*** | | ***TAT 2 (Sample reception Vs results dispatch)*** | | ***TAT 3 (Results dispatch Vs Results Receipt by Provider*** | | ***TAT 4***  ***(Results receipt by provider Vs Results Receipt by Mother*** | | ***TAT 5 (Sample Collection Vs Results Receipt by Mother)*** | |
| --- | --- | --- | --- | --- | --- | --- | --- | --- | --- | --- |
|  | ***N*** | ***%*** | ***N*** | ***%*** | ***N*** | ***%*** | ***N*** | ***%*** | ***N*** | ***%*** |
| ***Same day*** | ***175*** | ***96.7%*** | ***180*** | ***99%*** | ***140*** | ***77%*** | ***167*** | ***92%*** | ***111*** | ***61%*** |
| ***1 to 2*** | ***2*** | ***1.1%*** |  | ***0%*** | ***34*** | ***19%*** | ***9*** | ***5%*** | ***49*** | ***27%*** |
| ***3 to 4*** | ***1*** | ***0.6%*** | ***1*** | ***1%*** | ***3*** | ***2%*** | ***2*** | ***1%*** | ***12*** | ***7%*** |
| ***5 to 6*** | ***1*** | ***0.6%*** |  | ***0%*** | ***2*** | ***1%*** | ***1*** | ***1%*** | ***5*** | ***3%*** |
| ***7 to 8*** | ***1*** | ***0.6%*** |  | ***0%*** | ***1*** | ***1%*** | ***1*** | ***1%*** | ***2*** | ***1%*** |
| ***11 to 12*** | ***0*** | ***0.0%*** | ***0*** | ***0%*** | ***1*** | ***1%*** | ***1*** | ***1%*** | ***1*** | ***1%*** |
| ***Above 14*** | ***1*** | ***0.6%*** | ***0*** | ***0%*** | ***0*** | ***0%*** | ***0*** | ***0%*** | ***1*** | ***1%*** |
| ***Total*** | ***181*** | ***100.0%*** | ***181*** | ***100%*** | ***181*** | ***100%*** | ***181*** | ***100%*** | ***181*** | ***100%*** |
