# Supplementary material for: Optimizing hepatitis B diagnosis for mothers in a low-resource setting: A field pilot of Xpert point-of-care viral load testing in Ugandan antenatal clinics
Source: PLOS Glob Public Health. 2026 May 4;6(5):e0006380. doi: 10.1371/journal.pgph.0006380 (PMC13138665; doi:10.1371/journal.pgph.0006380)
Supplement: S4 File — (DOCX) [file pgph.0006380.s004.docx]

*Supplementary material 4: Itemised Costs for HBV VL Testing: COBAS vs GeneXpert Platforms*

| Category | COBAS Platform | Unit Cost (USD) | GeneXpert Platform | Unit Cost (USD) |
| --- | --- | --- | --- | --- |
| Main Assay Kit | KIT COBAS 5800 HBV 192T IVD | 8.78 | Xpert® HBV Viral Load (10 tests) | 14.90 |
| Controls | HBV/HCV/HIV-1 CONTROL CE-IVD (8 x 0.65 mL) | 1.85 | Built-in Internal Control | - |
| Negative Control | NHP NEG RMC IVD (16 x 1 mL) | 0.25 | Included | - |
| Processing Plates | COBAS OMNI PROCESSING PLATE | 0.07 | Not required | - |
| Amplification Plates | COBAS OMNI AMPLIFICATION PLATE | 0.19 | Not required | - |
| Wash Reagent | COBAS WASH Reagent IVD (4.2 L) | 0.02 | Included in the cartridge | - |
| Specimen Diluent | SPEC DIL REAGENT IVD (4 x 875 mL) | 0.42 | Included in the cartridge | - |
| Lysis Reagent | LYS REAGENT IVD (4 x 875 mL) | 0.34 | Included in the cartridge | - |
| Magnetic Particles | MGP IVD | 0.05 | Included in the cartridge | - |
| Pipette Tips | OMNI PIPETTE TIPS | 0.36 | Not required | - |
| Waste Bags | Solid Waste Bag Set of 20 | 0.16 | Cartridge Disposal | unknown |
| Secondary Tubes | Secondary tubes (Box of 1000) | 0.29 | Basic pipette only | unknown |
| Total Assay-Cost |  | 12.79 |  | 14.90 |
| Phlebotomy Supplies | Blood Collection Tube (K2-EDTA + PPT) | 0.15 | Same | 0.15 |
|  | Needle & Tube Holder (21G, 1.25 in) | 0.27 | Same | 0.27 |
|  | Gloves | 0.05 | Same | 0.05 |
| Total Phlebotomy Cost |  | 0.47 |  | 0.47 |
| Sample Transportation | **Cost per sample** | **2.0** | Test done onsite |  |
| The overall cost per test |  | **15.26** |  | **15.37** |
