# Supplementary material for: Optimizing hepatitis B diagnosis for mothers in a low-resource setting: A field pilot of Xpert point-of-care viral load testing in Ugandan antenatal clinics
Source: PLOS Glob Public Health. 2026 May 4;6(5):e0006380. doi: 10.1371/journal.pgph.0006380 (PMC13138665; doi:10.1371/journal.pgph.0006380)
Supplement: Attachment — Submitted filename: Response to Editors and Reviewers -HBV.docx [file pgph.0006380.s007.docx]

**PGPH-D-25-02257**

**Optimizing Hepatitis B Diagnosis for Mothers in a Low-Resource Setting: A Field Pilot of Xpert® Point-of-Care Viral Load Testing in Ugandan Antenatal Clinics**

**Short title: Xpert® Point of Care Viral Load Testing for Hepatitis B in Antenatal Clinics**

We thank the Editor and Reviewers for their comprehensive and thoughtful feedback, which has been invaluable in strengthening the clarity, rigor, and overall quality of the manuscript. The comments provided have enabled us to substantially improve the presentation, interpretation, and contextualization of our findings.

Below, we provide a detailed, point-by-point response to the Editor’s and Reviewers’ comments. All comments are reproduced verbatim to ensure transparency, and each concern is addressed in the corresponding response column, with revisions to the manuscript described where applicable.

| **Editor / Reviewer Comments** | **Response to the Editors and Reviewers** |
| --- | --- |
| **Additional Editor Comments (if provided):** *Cohen's Kappa (κ) statistics can be used for inter-assay reliability.* | We thank the Editor for this helpful suggestion. Given that viral load measurements in this study were continuous (See Supplementary material 1) rather than categorical, and that our primary objective was to assess agreement between two quantitative assays and their clinical performance at a relevant treatment threshold, we used Bland–Altman analysis to evaluate agreement across the measurement range. In addition, sensitivity and specificity were assessed at >20,000 IU/mL against the reference assay. Cohen’s kappa, which is typically applied to categorical outcomes, was therefore not used in this analysis. |
| *The study should also highlight turnaround time differences between the assays, if any, impacting actual patient care and outcomes* | We appreciate this comment. There were no assay-specific processing time differences that would meaningfully affect patient care once samples entered the analytical phase. The centralized COBAS platform is a high-throughput, fully automated system capable of processing up to 90 samples within approximately four hours, while the GeneXpert platform has lower throughput (typically 8–16 modules) but generates results within about one hour per run and requires manual sample preparation. In practice, these analytical time differences largely offset each other.  The primary difference affecting patient care lies in test placement rather than analytical speed. GeneXpert platforms are decentralized and available at or near the point of care, whereas the COBAS platform is located at the central public health laboratory. Consequently, delays in the centralized model arise mainly during the pre-analytical phase, including sample collection, packaging, transport, receipt, and logging, as well as results return which are avoided with point-of-care testing.  **We have revised the discussion to capture this perspective: - Line 324 - 327** *While analytical processing times for GeneXpert and centralized COBAS platforms are broadly comparable, the decentralized placement of GeneXpert at the point of care or near point of care eliminates pre-analytical delays related to sample transportation, handling and results return, thus resulting in a substantially shorter turnaround time for clinical decisions.* |
| **Reviewer #1:** *1. The manuscript should more prominently highlight the substantial loss to prophylaxis follow-up (only 14 of the 22 pregnant women eligible for antiviral prophylaxis received it) and the absence of birth-record follow-up (birth records were available for only 15 of the 181 pregnant women tested). It is important to address the reasons for these gaps, as they significantly limit the ability to draw meaningful conclusions about the intervention’s effectiveness in improving overall maternal–infant care linkage. Additionally, please correct the statement in line 324: “we found that 15 of the 22 pregnant women eligible for prophylaxis had received it.” Based on the previously reported data, the number of women who received prophylaxis was 14.* | We thank the reviewer for this observation and agree that gaps in prophylaxis follow-up and birth-record documentation are important limitations. The study was designed as a pragmatic implementation study focused on the feasibility of linking HBsAg-positive pregnant women to molecular testing without any interventions to support active management of downstream services for antenatal, maternity, or postnatal care, in line with routine Ministry of Health practice.  Active follow-up was therefore limited to ensuring that the 22 women eligible for prophylaxis were followed, rather than all 181 women screened. Nevertheless, the fact that only 14 of 22 eligible women received women-initiated prophylaxis and that birth records were available for only 15 women, including missing records for 7 eligible women, highlights critical gaps in care continuity and documentation that limit conclusions on end-to-end maternal–infant care linkage.  These issues are now more clearly emphasized in the limitation section of the revised manuscript as key implementation limitations.  We have also corrected the statement in line 324.  ***See lines 434-443 for the revisions made*** |
| **Reviewer #1:** *2. The study was co-financed by Cepheid, the manufacturer of the Xpert® assay, which also supplied the test cartridges. It would be best practice to expand the discussion to clarify how potential conflicts of interest were managed and how objectivity was ensured.*  *Furthermore, does the reported cost per test (USD 15.37) account for indirect expenses such as essential equipment maintenance, long-term logistical support, and patient-incurred costs? While cartridges for this study were provided by the manufacturer, it would be useful to discuss the long-term sustainability and cost implications beyond the study period.* | We thank the reviewer for this important comment. While the study was co-financed by Cepheid, including the provision of Xpert® test cartridges, the manufacturer had no role in study design, implementation, data collection, analysis, interpretation, or manuscript preparation. All analyses were conducted independently by the study team, and results are reported irrespective of direction or magnitude. We have expanded the manuscript to explicitly clarify how potential conflicts of interest were managed and how scientific objectivity was ensured.  ***Conflict of Interest Statement: Line 459-467***  *The study was co-financed by Cepheid, which also supplied the Xpert® test cartridges. Cepheid had no role in the study design, implementation, data collection, analysis, interpretation of results, or manuscript preparation. Only periodic progress updates and operational challenges related to kit supply were shared with the manufacturer; no study findings, analyses, or interim results were communicated. All analyses and reporting were conducted independently by the study team. Additionally, oversight was provided through institutional ethical review and adherence to approved study protocols, further safeguarding scientific independence.*  Equally, the reported cost per test (USD 15.37) reflects direct testing costs incurred during the study period and does not include indirect expenses such as equipment maintenance, long-term logistical support, or patient-incurred costs. As cartridges were provided by the manufacturer for this study, the estimate does not represent full programmatic costs beyond the study period. We have now clarified this in the Discussion by adding the following statement: **Line 366-369**  *We are cognizant that this costing was conducted from a provider perspective during the study period and does not represent a full economic or societal cost. Indirect expenses, including essential equipment maintenance, long-term logistical support, and patient-incurred costs, were not captured, but are likely lower than under the counterfactual.* |
| **Reviewer #2:** *The authors have conducted an extensive assessment of the functional feasibility of the Xpert® HBV Viral Load (VL) point-of-care assay in comparison with the reference COBAS® AmpliPrep/COBAS TaqMan® platform. However, the study lacks substantive scientific novelty. Both diagnostic kits have already undergone comprehensive validation and regulatory evaluation prior to market approval, and their analytical performance, accuracy, and intended use are clearly described in their respective manufacturer manuals.* | We agree with the reviewer that the analytical performance of both the Xpert® HBV Viral Load assay and the COBAS® AmpliPrep/COBAS TaqMan® platform has been previously established, as acknowledged in the manuscript (background, lines 90-100). The laboratory comparison served as both an in-country process for adopting new technology and as verification to provide confidence for its use in-country.  The purpose of this study was not to revalidate analytical accuracy but to document operational experience with the deployment of point-of-care HBV viral load testing within routine antenatal care services. In the absence of WHO prequalification and limited programmatic use to date, the study provides practical insights on workflow integration, turnaround time, care linkage, and cost considerations under real-world conditions. We have revised the manuscript discussion to more clearly emphasize this implementation and policy-relevant contribution. |
| **Reviewer #2:** *In this context, evaluating the operational feasibility of an already established and commercially validated diagnostic platform adds limited new scientific value. Operational feasibility is largely determined by broader programmatic and administrative factors, such as government policy, infrastructure availability, supply-chain efficiency, human-resource capacity, and national implementation strategy, rather than by laboratory-based comparative assessments alone. Without integrating these real-world determinants, the study’s contribution remains narrow.* | We thank the reviewer for this important perspective and agree that broader programmatic and administrative determinants, including policy, infrastructure, supply chain performance, human resource capacity, and national implementation strategies, shape operational feasibility. This study was not designed to comprehensively assess all these system-level factors. Rather, its scope was intentionally limited to documenting operational experience at the service-delivery level, focusing on how point-of-care HBV viral load testing functioned within existing antenatal care workflows under routine conditions. This is largely because there is strong programmatic experience on the service delivery gaps, amidst supportive national policies.  While we acknowledge that this provides only a partial view of operational feasibility, the study offers granular, practice-based insights into turnaround time, workflow integration, and linkage to clinical decision-making at the facility level, which are often underreported but necessary inputs for broader implementation planning. These also provide evidence for policy adoption and guidance on other programmatic and administrative adoptions.  We have revised the Discussion to more clearly situate the findings within these limitations and to emphasize that they should be interpreted as complementary to, rather than substitutes for, the system-level assessments required for national-scale up.  ***We have added some revisions to lines: 429-443***  *Furthermore, the findings should be interpreted within the broader operational constraints of delivering antenatal services in resource-limited settings, where feasibility is shaped not only by diagnostic performance but also by policy frameworks, infrastructure, supply-chain reliability, human-resource capacity, and routine documentation practices. While the study documented service-level experience with integrating hepatitis B viral load testing into routine antenatal care, gaps were observed in downstream care linkage and documentation. Of the 22 pregnant women eligible for antiviral prophylaxis, only 14 were documented to have initiated treatment, and birth records were available for only 15 women, including missing records for 7 women eligible for prophylaxis. These losses reflect the pragmatic design of the study, in which antenatal, delivery, and* *postnatal services followed routine standards of care without active follow-up by the study team. The incomplete initiation of prophylaxis and missing birth documentation highlight systemic weaknesses in care continuity, monitoring, and record-keeping that extend beyond the diagnostic intervention itself, which any country considering adoption needs to be aware of and program for. Addressing these broader programmatic and administrative factors is essential to realize the full potential of point-of-care viral load testing within maternal and child health services.* |
| **Reviewer #2:** *The work would have offered greater novelty if the authors had developed: 1) Modified or optimized methods using these existing kits tailored for resource-constrained or operationally challenging hospital settings; or 2) Cost-effective alternative workflows or algorithms that improve accessibility or turnaround time beyond standard manufacturer recommendations.* | We thank the reviewer for this constructive suggestion and note that the study was designed to address these issues at an operational level.  Although the GeneXpert platform is widely available in Uganda, its use has historically been limited to HIV, tuberculosis, HPV, and COVID-19 testing, and this study represents the first documented use of the Xpert® HBV Viral Load assay within antenatal care settings in the country. The selected facilities, while high-volume regional referral hospitals, face substantial operational challenges that have resulted in limited access to HBV viral load testing and delayed initiation of prophylaxis, despite national policies promoting integrated testing and treatment under the triple elimination strategy.  The study was therefore premised on the hypothesis that reliance on centralized viral load testing constituted a key bottleneck to timely eligibility assessment for prophylaxis, and that introducing point-of-care testing could improve access and turnaround time while holding other system factors constant. While we did not modify the assay itself, the operational experience generated provides practical evidence to inform context-appropriate adaptations to HBV testing algorithms, workflow integration, and programmatic planning, including supply allocation, in resource-constrained antenatal care settings.  ***We have updated the discussion to capture this perspective, Lines 407-414*** |
| **Reviewer #2:** *Another potential area of novelty could have been evaluating clinical impact, for example, assessing whether diagnosing pregnant women with these assays leads to measurable improvements in HBV prophylaxis outcomes in newborns. Such analyses would extend the study beyond analytical comparison toward clinically meaningful and policy-relevant insights.* | We agree that evaluating neonatal prophylaxis outcomes would add clinical depth. However, this was beyond the scope of the current study, which focused on documenting operational experience with integrating point-of-care HBV viral load testing into routine antenatal care and its implications for timely eligibility assessment for maternal prophylaxis. In addition, neonatal prophylaxis outcomes in Uganda have been documented in prior studies, and replicating these analyses was unlikely to yield substantially new insights within the context of this work. |
| **Dear Authors***, thank you for submitting your manuscript. I found your study to be relevant, given the ongoing challenges in scaling up hepatitis B diagnosis and prevention in low-resource settings. The findings on decentralized testing with GeneXpert are timely and could inform practice in Uganda and beyond.* | We thank the reviewer for this positive assessment and for recognizing the relevance of decentralized HBV testing in low-resource settings. |
| **Reviewer #3:** *To further strengthen your manuscript, I suggest clarifying the rationale for selecting high-volume hospitals and discussing how this might affect generalizability. It would also be helpful to expand on the diagnostic accuracy of the Xpert® assay, including the limit of detection and any discrepant results. More detail on the process for obtaining informed consent, as well as reasons for non-uptake of prophylaxis, would add depth. Discussing barriers to full uptake of prophylaxis and newborn vaccination, with references to local or regional studies, would strengthen the context. Finally, addressing the generalizability of your findings to other low-resource settings and acknowledging limitations like sample size and selection bias, would help readers better interpret your results.*  *With these improvements, your paper will be a valuable contribution to the literature on hep B prevention and point-of-care diagnostics in resource-limited settings.* |  |
|  | We thank the reviewer for these helpful comments and have revised the manuscript accordingly. We now clarify the rationale for selecting high-volume regional referral hospitals as primary HBV PMTCT referral sites with existing laboratory infrastructure, and we discuss implications for generalizability. We expanded the Discussion to summarize the established diagnostic accuracy and limits of detection of the Xpert® HBV Viral Load assay and to contextualize the limited discrepant results observed.  We also added detail on the informed consent process and strengthened the discussion of reasons for non-uptake of prophylaxis and incomplete infant follow-up, drawing on local and regional evidence on financial, counselling, and health system barriers. Finally, we more explicitly acknowledge limitations related to sample size, site selection, and scope, and discuss the applicability of findings to other low-resource settings with comparable capacity.  ***See discussion section*** |
