# Supplementary material for: Optimizing hepatitis B diagnosis for mothers in a low-resource setting: A field pilot of Xpert point-of-care viral load testing in Ugandan antenatal clinics
Source: PLOS Glob Public Health. 2026 May 4;6(5):e0006380. doi: 10.1371/journal.pgph.0006380 (PMC13138665; doi:10.1371/journal.pgph.0006380)
Supplement: Attachment — Submitted filename: v1 Response to Editors and Reviewers 7th April.docx [file pgph.0006380.s008.docx]

**Response to Editors and Reviewers**

| **#** | **Comment** | **Response** |
| --- | --- | --- |
| **JOURNAL REQUIREMENTS** | | |
| 1a | We have evaluated Figures 1 and 2 and the Supporting Information files you have provided. While Figure 2 is acceptable - as it includes only generic shapes - Figure 1 and Figure 1 and "Xpert HBV Viral Load ENGLISH IFU 301-5878 Rev. F (1).pdf" contain copyrighted material that cannot be published in PLOS. | We have edited Fig 1. All icons have been removed.   The PDF is for attachment to the portal, while the Image is for copying and pasting to the manuscript. |
| 1b | Additionally, we note that your Supporting Information file "Xpert HBV Viral Load ENGLISH IFU 301-5878 Rev. F (1).pdf" contains numerous logos, potentially copyrighted images, and potentially copyrighted software User Interfaces. Please note that PLOS cannot publish any logos of any kind. | We have removed the product information document. |
| 2 | Please provide separate main figure files in .tif or .eps format only, and ensure that all files are within our 10 MB size limit. | These have been provided. |
